# The potential role of heparin-binding protein in neonatal sepsis: research progress

**DOI:** 10.3389/fcimb.2024.1422872

**Published:** 2024-08-13

**Authors:** Xiyang Chen, Haiting Li, Jie Li, Xixi Liu, Linlin Chen, Caie Chen, Junhui Yuan, Enfu Tao

**Affiliations:** Department of Neonatology and Neonatal Intensive Care Unit, Wenling Maternal and Child Health Care Hospital, Wenling, Zhejiang, China

**Keywords:** heparin-binding protein, neonatal sepsis, biomarker, early diagnosis, therapeutic intervention

## Abstract

Neonatal sepsis is a major global health challenge, leading to significant morbidity and mortality in newborns. The search for precise biomarkers for its early prediction in clinical settings has been ongoing, with heparin-binding protein (HBP) emerging as a promising candidate. Originating from granules in neutrophils, HBP is released into the bloodstream in response to infection and plays a pivotal role in the body’s inflammatory response. Its significance extends beyond its inflammatory origins; research indicates dynamic changes in HBP levels are strongly linked to reduce in-hospital mortality, offering a prognostic advantage over existing biomarkers. Furthermore, HBP has demonstrated considerable clinical utility in the early diagnosis and stratification of neonatal sepsis, suggesting its potential as a reliable blood marker for early prediction of the disease and its severity. Its application may extend to guiding the judicious use of antibiotics in treating newborns, addressing a critical aspect of neonatal care. Despite these encouraging results, the precise clinical utility of HBP for diagnosing and treating sepsis in neonates still demands further clarification through extensive research. This review delves into the current scientific understanding of HBP’s contribution to diagnosing, prognosticating, and treating neonatal sepsis, while considering its future clinical applications.

## Introduction

Neonatal sepsis is a systemic inflammatory response caused by bacterial infection and is a leading cause of morbidity and mortality in newborns. It also represents an urgent health issue worldwide (Kim et al., 2020; Milton et al., 2022). The incidence is approximately 2.2%, with mortality rates between 11% and 19% (Fleischmann-Struzek et al., 2018). In high-income countries, neonatal sepsis occurs in 1 to 4 per 1,000 live births, while in middle- to low-income countries, it is significantly higher at 46.9-170 per 1,000 live births, with a mortality rate up to 24% (Celik et al., 2022; Milton et al., 2022). Early diagnosis and treatment are crucial to prevent severe complications and reduce mortality. Despite a decrease in incidence due to the prophylactic use of antibiotics (Puopolo et al., 2017; Fleiss et al., 2023), ideal early diagnostic markers are still lacking. Blood culture results take about 21 hours (Kuzniewicz et al., 2020), while waiting for blood culture results, 2% to 15% of full-term newborns and 87% to 96% of extremely preterm infants (gestational age ≤25 weeks or birth weight <1000 grams) admitted to neonatal intensive care units are treated with antibiotics (Oliver et al., 2017; Puopolo et al., 2017; Flannery et al., 2018). Rapid and accurate biomarkers are essential, as delayed treatment may increase the risk of death from septic shock (Iregbu et al., 2022). Although numerous biomarkers have been proposed in the field of sepsis, none have been deemed sufficient for routine clinical application (Pierrakos et al., 2020; Liu et al., 2023).

Commonly infection markers in clinical settings like C-reactive protein (CRP) and procalcitonin (PCT) (Stocker et al., 2017) show increased responses to non-infectious stimuli within the first 48 hours of birth, making them unsuitable for early-onset sepsis (EOS) diagnosis. However, they have good negative predictive accuracy within 36 hours, aiding in ruling out sepsis (Stocker et al., 2021). Other markers, including white blood cell (WBC) levels and presepsin (Poggi et al., 2022), interleukin (IL)-6 (Küng et al., 2023)and IL-8 (Ahmed et al., 2019), point-of-care testing for serum amyloid a (POC-SAA) (Sharma et al., 2023), mean platelet volume (MPV) (Toro-Huamanchumo et al., 2022), platelet to lymphocyte ratio (PLR) (Arcagok and Karabulut, 2019), progranulin (PGRN) (Yang et al., 2020), carboxyhemoglobin (COHb) levels (Dani et al., 2023), prognostic nutritional index (PNI) (Li et al., 2021b), procalcitonin-to-albumin ratio (PAR) (Li et al., 2022), CD11b (Qiu et al., 2019), CD64 (Shi et al., 2016), and soluble triggering receptor expressed on myeloid cells-1 (sTREM-1) (Bellos et al., 2018), lack large-scale studies, and fail to address antibiotic use challenges (Stocker and Giannoni, 2024). Recently, heparin-binding protein (HBP) has gained attention for its role in inflammation and infection. HBP exhibits strong antimicrobial and pro-inflammatory activities, inducing vascular leakage and attracting neutrophils, monocytes, and T lymphocytes, making it an important predictor for sepsis and organ dysfunction (Fisher and Linder, 2017).

This review aims to explore the application value of HBP in neonatal sepsis, focusing on its potential as a biomarker and therapeutic tool. We will review current scientific literature, analyze the role of HBP in the diagnosis, prognosis assessment, and treatment of neonatal sepsis, and discuss its prospects for clinical application.

## Pathophysiological mechanisms of neonatal sepsis

Neonatal sepsis is a nonspecific inflammatory response that involves complications across multiple systems (Li et al., 2021a). It is primarily caused by the transmission of microbes into the neonate’s bloodstream, triggering an immune response that can lead to a systemic inflammatory response syndrome (SIRS), potentially progressing to sepsis, multiple organ failure, and death (Akhmaltdinova et al., 2022). The underdeveloped neonate’s innate immune system increases the risk of sepsis, as neonates have hypogammaglobulinemia due to the lack of IgA and IgM transfer through the placenta. IgG transfer begins around the 13th week of pregnancy, reaching 10% of maternal levels by week 22, 50% by week 32, and peaking in the last four weeks of gestation. Preterm infants born before 36 weeks have insufficient plasma IgG levels, increasing sepsis risk (Beudeker et al., 2022).Neonates experience a physiological reduction in neutrophil count, and their migration and phagocytic capabilities are impaired (Arcagok and Karabulut, 2019). Studies show the neonatal immune system responds differently to bacterial antigens, producing lower levels of tumor necrosis factor-alpha (TNF-α) and higher levels of IL-6 compared to adults (Kurul et al., 2022). The pathophysiology of sepsis involves both hyperinflammatory syndrome and immunosuppression. The hyperinflammatory response, manifesting as shock and fever, is primarily driven by leukocytes, cytokines, reactive oxygen species, endothelial cells, the complement and coagulation systems. While the local activation of these pro-inflammatory and pro-coagulant mechanisms following infection is part of the protective innate immune response, their uncontrolled activity can lead to collateral damage and plays a key role in the pathogenesis of sepsis (Wiersinga and van der Poll, 2022). The uncontrolled activity of pro-inflammatory cytokines, such as TNF and IL-1β, is believed to play a significant role in tissue damage (Wiersinga and van der Poll, 2022). Sepsis-associated immunosuppression involves various cell types and is characterized by immune cell exhaustion, cellular apoptosis, T-cell depletion, and the reprogramming of antigen-presenting cells. This condition results in a diminished capacity to produce pro-inflammatory cytokines upon stimulation and a reduction in the expression of cell surface molecules, such as HLA-DR (Torres et al., 2022). Increased expression of the inhibitory co-stimulatory molecules PD-1 and its ligand PD-L1 on circulating monocytes, neutrophils, and effector T cells leads to sepsis-induced immunosuppression (Hibbert et al., 2018). The role of the PD-1/PD-L1 pathway in neonates is not yet fully understood, but it is known to play a crucial role in regulating immune responses by reducing T cell activity to prevent autoimmune tissue damage (Akhmaltdinova et al., 2022). Additionally, another key characteristic of sepsis is the increased vascular permeability, leading to edema, hypotension, and inadequate organ perfusion. The release of inflammatory mediators can damage the integrity of the endothelial wall by disrupting intercellular tight junctions, leading to endothelial leakage and the subsequent movement of fluids into the extravascular space, decreasing circulating blood volume and subsequently reducing cardiac preload. Inflammation also damages the endothelial glycocalyx and releases nitric oxide (NO) and endothelins, two major mediators of vascular tone, altering blood flow to organs. This hypotension and inadequate organ perfusion can rapidly progress to shock, posing a significant threat to neonatal survival (Kharrat and Jain, 2022). Sepsis also involves coagulation dysfunction, where platelets play a significant role in the coagulopathy induced by sepsis (Arcagok and Karabulut, 2019).

## Early-onset sepsis and diagnostic biomarkers

Neonatal sepsis is categorized into early-onset sepsis (EOS) and late-onset sepsis (LOS) (Shane et al., 2017). EOS refers to the growth of pathogenic microorganisms in blood or cerebrospinal fluid cultures obtained within the first 72 hours of life (Poggi et al., 2022). EOS is acquired prenatally or during delivery and typically represents vertical transmission from mother to infant (Shane et al., 2017). The risk of EOS is inversely related to gestational age, with the highest incidence in infants born between 22 and 28 weeks of gestation (18.47 per 1000 live births) and the lowest in full-term infants (0.5 per 1000 live births) (Fleiss et al., 2023). Studies have reported that the mortality rate for EOS is 18%, with higher rates in preterm infants, those with septic shock, and those requiring mechanical ventilation (Giannoni et al., 2018). Early antibiotic use is critical for prognosis (Stocker et al., 2021). Group B *Streptococcus* (GBS) and *Escherichia coli* (*E. coli*) cause nearly 70% of EOS cases with *E. coli* being the leading cause of morbidity and mortality (Glaser et al., 2021). EOS progresses rapidly, potentially leading to multi-organ failure and death (Chinese Maternal and Child Health Association Neonatal Healthcare Professional Committee and C.M.D.A.N, 2021). Clinical symptoms of EOS, including fever, rapid breathing, lethargy, and poor feeding, often overlap with normal newborn physiological changes, making diagnosis challenging (Kim et al., 2020; Chinese Maternal and Child Health Association Neonatal Healthcare Professional Committee and C.M.D.A.N, 2021; Poggi et al., 2022; Fleiss et al., 2023). PCT and CRP are currently the most commonly used biomarkers but can produce false-positive results, due to the physiological response in the first 48 hours of life (Memar et al., 2019; Tiozzo and Mukhopadhyay, 2022). A focused study evaluated the diagnostic precision of sequential assessments of CRP, PCT, and WBC in excluding EOS within a 36-hour timeframe. Findings demonstrated that, within this period, the peak values of CRP, PCT, and WBC, when compared to non-septic instances, achieved area under the curve (AUC) values of 0.986, 0.921, and 0.360, respectively, effectively distinguishing verified sepsis cases. Threshold values were determined at 16mg/L for CRP and 2.8ng/L for PCT, providing a sensitivity of 100% for discerning between the absence of sepsis and confirmed diagnoses (Stocker et al., 2021). IL-6 is the most effective biomarker for assessing EOS prognosis with a pooled sensitivity of 76% and specificity of 79% (Memar et al., 2019; Eichberger and Resch, 2022). Elevated serum levels of sTREM-1 and IL-6 are significantly in infected neonates, though sTREM-1’s diagnostic value does not surpass IL-6 alone (Sarafidis et al., 2010). The analysis on sTREM-1 included data from eight studies with a cohort of 667 neonates. It reported a sensitivity of 0.95 [95% CI (0.81-0.99)] and a specificity of 0.87 [95% CI (0.56-0.97)]. The diagnostic odds ratio was calculated at 132.49 [95% CI (6.85-2560.70)]. However, the limited number of studies and the variation in cutoff values restrict its application in clinical practice (Bellos et al., 2018). Another study evaluated PGRN as a new biomarker for EOS diagnosis in 121 infants with a gestational age over 34 weeks. Findings revealed significantly higher serum PGRN levels in the infected group (median 47.72 vs. 37.86 ng/ml) compared to the uninfected group. PGRN demonstrated a promising diagnostic potential with an area under the ROC curve of 0.786, a sensitivity of 94.34%, and a negative predictive value of 91.7%. Notably, PGRN, independently and in combination with PCT, significantly improved EOS diagnosis, achieving a specificity of 89.06% and a positive predictive value of 81.10%.The limitations of this study include the relatively small difference in PGRN levels between the infected and uninfected groups observed in this small, single-center study (Rao et al., 2020). A meta-analysis (Poggi et al., 2022) on presepsin involving 12 studies and 828 newborns of any gestational age revealed that the pooled sensitivity and specificity were 0.93 (95% CI, 0.86-0.95) and 0.91 (95% CI, 0.85-0.95), respectively, but the limitation is due to the small number of EOS cases included. CD11b has emerged as a promising biomarker for the early detection of neonatal sepsis. A meta-analysis demonstrated its high overall pooled sensitivity (0.82) and specificity (0.93) (Qiu et al., 2019). However, studies indicate that CD11b alone is not ideal and should be used in combination with other markers to enhance diagnostic accuracy (Qiu et al., 2019). A systematic review and meta-analysis evaluated the performance of neutrophil CD64 (nCD64) in diagnosing neonatal sepsis. Data from 17 studies involving 3478 participants showed an overall pooled sensitivity of 77% and specificity of 74%, indicating its high diagnostic accuracy. The AUC was 0.8666, further supporting its diagnostic value. However, the analysis cautioned that nCD64’s sensitivity and specificity are less than ideal when used alone (Shi et al., 2016). In another study, it was found that serum levels of nCD64, PCT, CRP, and WBC were significantly higher in the neonatal sepsis group compared to the non-sepsis group (p<0.001). At recommended cutoff points, the sensitivities of nCD64, PCT, CRP, and WBC were 79.5%, 68.2%, 38.6%, and 52.3%, respectively. The best combination was nCD64 and PCT, with a sensitivity of 90.9%, a maximum AUC of 0.922, and a negative predictive value of 89.2% (Yang et al., 2016).

## Late-onset sepsis and diagnostic biomarkers

LOS refers to a delayed infection caused by pathogens acquired after childbirth, typically beyond 3 to 7 days, due to exposure in the hospital or community environment (Shane et al., 2017). Moreover, later instances of LOS can occur at any time within the first three months (Kim et al., 2020).With advancements in the treatment of preterm infants and the increased survival rates of extremely low and very low birth weight infants, LOS has become more common (Dong et al., 2019).Studies from both developed and developing countries report that extremely low birth weight infants may account for 50% to 70% of neonatal LOS cases (Dong et al., 2019). LOS is an independent risk factor for mortality in extremely preterm and late preterm infants, leading to prolonged hospital stays (on average, more than 3 weeks) for very low birth weight neonates, neurodevelopmental impairments, growth deficiencies, and a mortality rate as high as 24% (Afonso et al., 2023). LOS is most commonly caused by Gram-positive bacteria, with the most frequent Gram-positive LOS pathogens including coagulase-negative staphylococci (50% of cases), *Staphylococcus aureus* (7% of cases), and GBS (1% of cases). Gram-negative bacteria account for 20% to 42% of LOS cases, including *E. coli*, *Klebsiella pneumoniae*, *Serratia marcescens*, *Enterobacter*, and *Pseudomonas aeruginosa*. *E. coli* is the most common Gram-negative bacterium, while *Pseudomonas aeruginosa* is the most lethal (Glaser et al., 2021). In most cases of LOS, the initial clinical symptoms are subtle and nonspecific, such as feeding difficulties, temperature changes, or respiratory instability. However, the disease course can be fulminant, leading to death within a matter of hours (Kurul et al., 2022). Currently, there are no accurate biomarkers available for the early prediction of LOS. Research by Brown and colleagues has found that assessing serum CRP levels in infants suspected of LOS does not aid in early diagnosis (Brown et al., 2020). A study indicated that within the LOS group, there were no differences in PCT, CRP, and PLT counts between the bacteremia and non-bacteremia groups. Only WBC counts with a cutoff value of 12.1×10^9^/L could be used as a predictive marker for bacteremia (Tang et al., 2022). sTREM-1 has demonstrated diagnostic accuracy in LOS. A study showed that sTREM-1 has potential as a predictor of septic shock and death in late-onset neonatal sepsis (LONS) patients. ROC curve analysis, using a suggested sTREM-1 cutoff value of 300 pg/mL, revealed an area under the curve of 0.884 (95% CI=0.73 to 1.0; p<0.0001), with a sensitivity of 0.78 (95% CI=0.46 to 0.94) and specificity of 0.97 (95% CI=0.92 to 0.99). However, due to sample limitations, larger-scale studies are needed to validate these findings (Arízaga-Ballesteros et al., 2015).Therefore, for both EOS and LOS, there is an urgent need for more precise infection markers to enable early diagnosis and treatment, thereby reducing mortality and avoiding adverse outcome.

## The physiological properties of heparin-binding protein and its role in inflammatory responses

**Figure 1:**
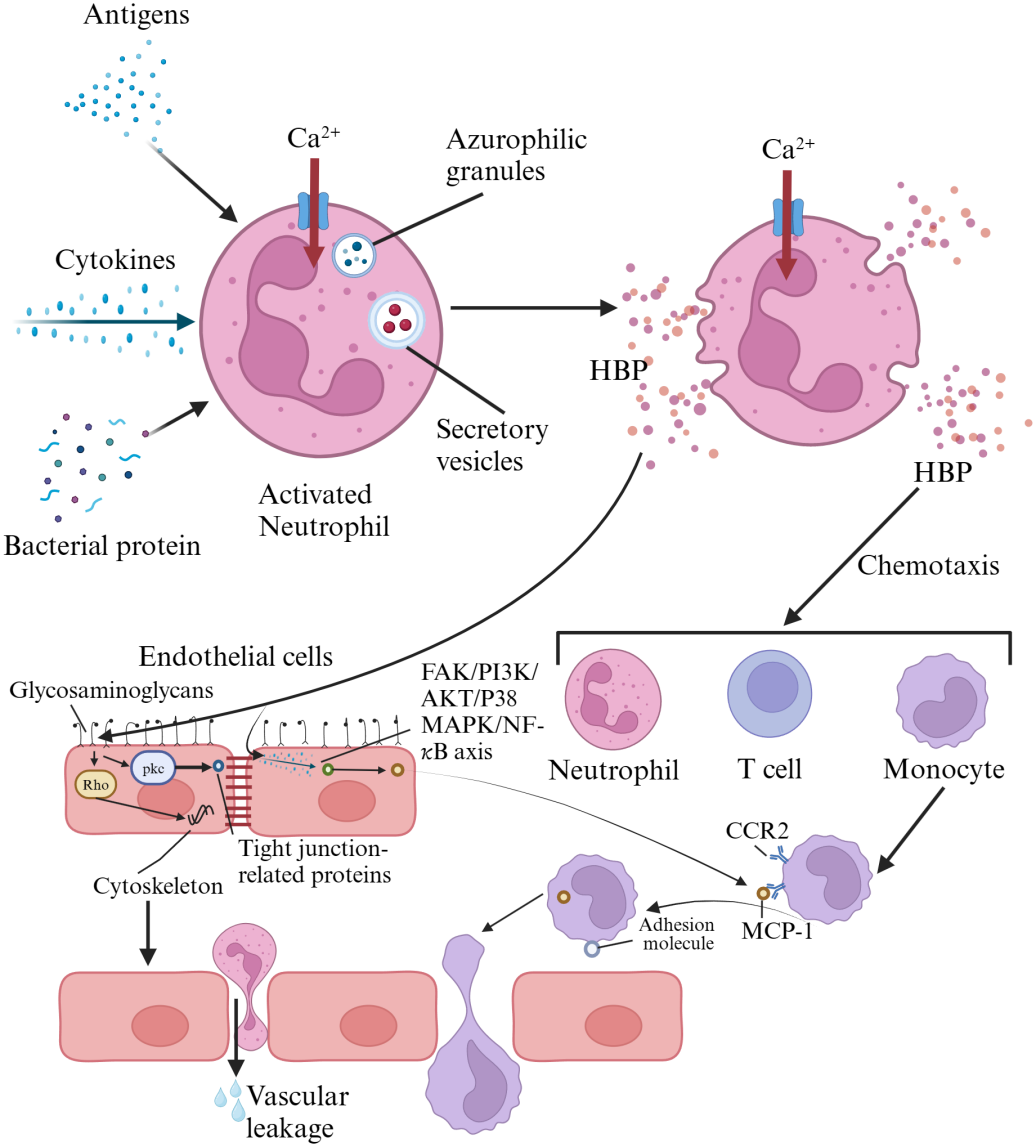
Mechanisms of heparin-binding protein action in inflammatory responses. HBP is pre-formed and released by activated neutrophils via secretory vesicles. HBP directly interacts with endothelial cells by binding to glycosaminoglycans, activating protein kinase C and Rho kinase. This triggers calcium influx, leading to cytoskeletal reorganization and cell contraction, creating gaps in endothelial cells that result in vascular leakage and facilitate neutrophil extravasation. HBP also induces the expression of monocyte chemoattractant protein-1 in vascular endothelial cells through the FAK/PI3K/AKT and p38 MAPK/NF-κB signaling pathways, promoting monocyte migration. HBP, heparin-binding protein; FAK, focal adhesion iinas; ‌PI3K, ‌phosphatidylinositol 3-kinase; CCR2, chemokine receptor 2; MCP-1, monocyte chemotactic protein 1.

HBP, also known as azurocidin, azurocide, or cationic antimicrobial protein of 37,000 Daltons (CAP37), was first isolated from neutrophils by Shafer et al. in 1984. It was named for its strong capacity to bind to heparin (Shafer et al., 1984). It is a newly discovered infection marker and a member of the serine protease family within the polymorphonuclear neutrophil lineage, primarily located in the secretory granules and azurophil granules of neutrophils (Fisher et al., 2022). Heparin acts as a ligand for HBP, binding to the N-terminal region of HBP, which consists of at least two clusters of basic residues. Once HBP binds to heparin, its antimicrobial activity is inhibited. However, the domains of HBP capable of binding to other cellular receptors remain exposed even after binding to heparin (Yang et al., 2019a). HBP is pre-formed and released by secretory vesicles from activated neutrophils, making it one of the earliest inflammatory mediators released in response to infection. Therefore, it serves as an excellent early predictive marker for sepsis (Fisher and Linder, 2017; Ren et al., 2021). When neutrophils in the blood are stimulated by infection or other factors, the release of HBP increases (Deng et al., 2018). The released HBP is primarily taken up by the liver and associates with hepatocytes, with a smaller amount being absorbed by monocytes/macrophages in the spleen (Fisher et al., 2022). HBP serves as a chemotactic factor for neutrophils, T cells, and monocytes, and it also enhances the release of cytokines by monocytes, their phagocytic activity, and adherence to endothelial cells. It possesses functions such as increasing vascular endothelial permeability, bactericidal chemotaxis, and regulating apoptosis (Fisher and Linder, 2017; Ren et al., 2017). The release of HBP has been confirmed in various infectious diseases, such as meningitis, pneumonia, and urinary tract infections. HBP has also been shown to be associated with neutrophil-induced vascular leakage (Liu et al., 2023). HBP interacts directly with endothelial cells by binding to glycosaminoglycans. It activates protein kinase C (PKC) and rho kinase, inducing calcium influx into cells, leading to cytoskeletal reorganization and cell contraction. This creates gaps in endothelial cells, resulting in vascular leakage and facilitating the extravasation of neutrophils (Fisher and Linder, 2017). HBP can induce the expression of monocyte chemoattractant protein-1 (MCP-1) in vascular endothelial cells through the FAK/PI3K/AKT and p38 MAPK/NF-kB signaling pathways, promoting monocyte migration (Chang et al., 2018; Yang et al., 2019a). Reports suggest that HBP may possess the capability to cleave certain insulin-like growth factor binding proteins (IGFBP-1, IGFBP-2, and IGFBP-4), thereby regulating inflammation and wound healing (Yang et al., 2019a). Research indicates that HBP has a short circulation time in the blood, with a distribution half-life of less than 10 minutes and an elimination half-life between 1 to 2 hours (Fisher et al., 2022). HBP has pro-inflammatory and vascular permeability-inducing effects, which can be detrimental in conditions such as sepsis (Fisher and Linder, 2017). Therefore, the rapid clearance of HBP from the circulation may serve as a protective mechanism against these adverse effects (Fisher et al., 2022). HBP activates various cell types and may become a pathogenic factor in sepsis; thus, its measurement can predict downstream sequelae (Fisher and Linder, 2017). The mechanisms of HBP action in inflammatory responses was summarized in **Figure 1**.

## The clinical application value of heparin-binding protein in infectious diseases

Liu and colleagues (Liu et al., 2023) highlighted that a decline in HBP levels within 72 hours of admission is significantly associated with a reduction in in-hospital mortality. Moreover, the prognostic value of dynamic changes in HBP surpasses that of other biomarkers. HBP levels show a weak positive correlation with PCT, CRP, and WBC, and no correlation with lactate levels. Another study explored the predictive value of HBP in assessing the 30-day mortality rate of sepsis patients in intensive care units. Through a prospective observation of 206 patients, it was found that changes in HBP within 48 hours provide a high level of accuracy in predicting 30-day mortality. Incorporating this into clinical prediction models significantly enhances the accuracy of predictions (Dou et al., 2022). Scholars have suggested that HBP in plasma can serve as a new diagnostic biomarker for a range of conditions including sepsis, bacterial skin infections, acute bacterial meningitis, leptospirosis, protozoan parasitic infections, and even some non-infectious diseases (Yang et al., 2019a). The “Chinese guidelines for management of severe sepsis and septic shock 2014” (Chinese Society of Critical Care Medicine, 2015) states that HBP can serve as an early diagnostic marker for sepsis, especially for severe bacterial infections. Currently, HBP is widely used in the diagnosis of infectious diseases in children and the assessment of disease severity. Blood HBP levels are a good predictive indicator for the progression of community-acquired pneumonia in children to respiratory failure and sepsis (Huang et al., 2021). A comprehensive study evaluated the accuracy of cerebrospinal fluid HBP in diagnosing hospital-acquired meningitis and ventriculitis, comparing it against procalcitonin and lactate levels. The results demonstrated that HBP exhibits high sensitivity and specificity, marking it as a highly effective early diagnostic tool for these conditions. This underscores HBP’s potential as a superior biomarker in the clinical landscape for identifying serious infections within hospital setting (Kong et al., 2022). The detection of HBP in cerebrospinal fluid is also utilized in the diagnosis and prognosis of pediatric purulent meningitis (Zhou et al., 2019; Ren et al., 2021).

## The role of heparin-binding protein in early diagnosis and assessment of neonatal sepsis

Blood HBP is increasingly being applied in the early diagnosis of neonatal sepsis. Studies have shown that plasma HBP levels in neonates with severe sepsis and septic shock are significantly higher than those in the general sepsis group, with a statistically significant difference. The AUC for diagnosing neonatal sepsis and neonatal infections with HBP are 0.885 and 0.904, respectively, both higher than those for PCT and high-sensitivity C-reactive protein (hs-CRP). This suggests that HBP may be superior to PCT and hs-CRP in the early diagnosis and clinical grading of neonatal sepsis, indicating its potential for valuable clinical application (Deng et al., 2018). Wang and colleagues (Wang et al., 2016) explored the application value of HBP in the diagnosis of early neonatal bacterial infectious diseases. The results showed that the HBP concentration in the early neonatal bacterial infection group was significantly higher than that in the control group (P < 0.001). When the cut-off value was set at 20ng/ml, the sensitivity and specificity of HBP in diagnosing early neonatal bacterial infections were 87.7% and 75.4%, respectively; when the cut-off value was set at 30ng/ml, the sensitivity and specificity were 82.2% and 76.8%, respectively. Moreover, the AUCs of HBP, PCT, CRP, and WBC were 0.866 (0.821 ~ 0.911), 0.673 (0.606 ~ 0.704), 0.651 (0.579 ~ 0.722), and 0.399 (0.326 ~ 0.442), respectively, indicating that HBP had the highest AUC and its diagnostic accuracy was better than PCT, CRP, and WBC. Deng and his team (Deng et al., 2018) conducted a retrospective analysis on the clinical value of HBP for the early diagnosis of neonatal sepsis and the grading of its severity. They discovered that the levels of HBP, PCT, and hs-CRP were significantly higher in the general sepsis group, the severe sepsis group, and the septic shock group compared to the localized infection group and the non-infection group. Furthermore, the plasma HBP levels in the severe sepsis group [52.35 (33.65, 88.15) ng/ml] and the septic shock group [73.55 (60.61, 145.51) ng/ml] were significantly higher than in the general sepsis group [34.12 (23.04, 41.79) ng/ml]. However, there was no statistically significant difference in serum PCT and hs-CRP levels among the three groups. The AUC for HBP in diagnosing neonatal sepsis was 0.885, which was higher than that for PCT and hs-CRP. With a cut-off value of 28.0ng/ml, the sensitivity and specificity of HBP in diagnosing neonatal sepsis were 80.4% and 88.4%, respectively. This suggests that HBP is superior to PCT and hs-CRP in the early diagnosis and clinical grading of neonatal sepsis. Ren and colleagues (Ren et al., 2018) also found that the HBP levels in the neonatal sepsis group (64.41 ± 78.51) ng/ml were higher than those in the control group (31.97 ± 20.76) ng/ml (*P* < 0.05). However, the AUCs for diagnosing neonatal bacterial diseases with HBP and PCT were 0.683 and 0.869, respectively. This suggests that, as a single indicator, PCT is superior to HBP in diagnosis, which is inconsistent with the findings of Wang (Wang et al., 2016) and Deng (Deng et al., 2018). The discrepancy might be related to the smaller sample size in Ren’s study (Ren et al., 2018). In a multicenter cross-sectional study, patients with sepsis showed a significant increase in HBP levels, which were positively correlated with PCT, CRP, counts of neutrophils and monocytes, creatinine, bilirubin, and lactate. When the HBP level was > 19.8 ng/mL, the sensitivity, specificity, positive predictive value (PPV), and negative predictive value (NPV) for diagnosing sepsis were 66.3%, 44.9%, 49.3%, and 62.2%, respectively. For predicting early mortality, these values were 100%, 41.0%, 4.5%, and 100%, respectively. When HBP and PCT were used in combination, the sensitivity, specificity, PPV, and NPV were 44.8%, 81.8%, 17.3%, and 94.6%, respectively (Katsaros et al., 2022). A prospective cohort study highlighted that in the ROC curve for diagnosing sepsis, HBP had an AUC of 0.893. The optimal cutoff value was identified as HBP ≥ 28.1ng/mL, resulting in a sensitivity of 84.9%, specificity of 78.3%, PPV of 94.0%, and NPV of 65.9% for the diagnosis of sepsis. These metrics were superior to those of PCT, CRP, and the sequential organ failure assessment (SOFA) score. In the ROC curve for diagnosing septic shock, the HBP level was also identified as the best biomarker with an AUC of 0.760 (Zhou et al., 2019). Yang et al. (2019b) combined the SOFA score with HBP, reaching an AUC of 0.83. Compared to the AUC of 0.81 for HBP as a single marker, the combined approach showed a sensitivity of 92.9% and a specificity of 61.9%. However, these three studies were applicable only to adults, with a lack of corresponding data for neonates. In summary, HBP could be a reliable blood biomarker for early prediction of neonatal sepsis and the severity of the disease. Nonetheless, further clarification is required through large-scale prospective studies.

## The role of heparin-binding protein in guiding antibiotic usage

With the growing seriousness of antibiotic resistance, the rational use of antibiotics has become a global challenge. An international, cross-sector study involving 757,979 late preterm and full-term newborns from 13 networks across Europe, North America, and Australia found that 2.86% of the newborns received antibiotic treatment within the first week. The incidence of EOS was 0.49 cases per 1,000 live births, with an EOS-related mortality rate of 3.20%. The study highlighted that the use of antibiotics was excessively frequent compared to the burden of EOS, and there was up to a 9-fold difference in antibiotic use among different networks (Giannoni et al., 2022). The early empirical use of antibiotics without positive blood culture evidence is associated with adverse outcomes, such as LOS, necrotizing enterocolitis (NEC) (Dierikx et al., 2022), mortality (Kuppala et al., 2011), broncho pulmonary dysplasia (BPD) (Cantey et al., 2017), adverse neurodevelopmental outcomes (Slykerman et al., 2019), asthma (Renz and Skevaki, 2021), food allergies (Alm et al., 2014) and others. A multicenter cohort study found that nearly 90% of preterm infants received empirical antibiotic treatment within the first 72 hours after birth due to suspected EOS. One quarter of these infants underwent treatment for more than 72 hours. Short-term (≤72 hours) antibiotic treatment, compared to no antibiotic use and prolonged antibiotic therapy, was associated with an increased risk of NEC. On the other hand, prolonged empirical antibiotic exposure increased the risk of LOS (Dierikx et al., 2022). Research has revealed that a mere 48 hours of antibiotic treatment can profoundly alter the diversity and structure of the gut microbiome in infants, along with influencing the emergence of antimicrobial resistance genes. Remarkably, these changes were detectable for up to a year following birth, suggesting that short-term antibiotic exposure can have enduring effects on the evolving gut ecosystem of newborns (Reyman et al., 2022). Furthermore, an investigation into the effects of empirical antibiotic usage on the mortality rate and the development of BPD in infants with extremely low birth weight has demonstrated that, when accounting for illness severity, each additional day of antibiotic treatment correlates with an increased risk and severity of BPD (Cantey et al., 2017). Slykerman et al. (2019) conducted a study exploring the association between antibiotic treatment in the first 24 months of life and neurocognitive outcomes at the age of 11. The results indicated that children who received antibiotics within the first six months of life had significantly lower overall cognitive abilities and language comprehension skills. The detection of HBP offers a new strategy that can help physicians reduce unnecessary use of antibiotics in the absence of clear signs of bacterial infection. In one study, by measuring the levels of HBP in the blood of patients with acute infections, researchers were able to accurately distinguish between bacterial and non-bacterial infections. This provided timely and precise guidance for clinicians, aiding them in making more rational decisions regarding the use of antibiotics (Fisher et al., 2017). Utilizing HBP levels as a biomarker provides a novel approach for gauging infection risks in neonates. By closely monitoring HBP concentrations in their blood, physicians can gain deeper insights into the infection’s severity. This enables a more nuanced evaluation and allows for the fine-tuning of treatment strategies accordingly (Fisher and Linder, 2017). Given the current lack of large-scale, multicenter prospective studies on the early monitoring of HBP for predicting the value in EOS and LOS, as well as its potential to optimize the use of antibiotics and improve long-term outcomes in newborns, further research is warranted. This area of investigation presents a crucial opportunity to deepen our understanding of HBP’s role in neonatal care, offering the possibility of enhancing treatment precision and ultimately bettering neonatal health prospects.

## Conclusions and perspectives

Sepsis continues to pose a significant threat to the health and safety of newborns, with an ideal biomarker for its prediction still out of reach. As the search for a definitive biomarker for neonatal sepsis continues, HBP emerges as a promising candidate. Demonstrating significant clinical value in detecting bacterial infections in adults and children, HBP’s role in neonates has been corroborated by preliminary studies, indicating its potential in disease prediction, severity differentiation, and guiding antibiotic utilization. Nevertheless, the transition to widespread clinical adoption is contingent upon a stronger foundation of evidence-based medicine. The imperative for future research lies in conducting expansive, multicenter prospective studies to ascertain HBP’s efficacy in the early identification of EOS and LOS in newborns. Additionally, its influence on antibiotic stewardship and the association with long-term health trajectories warrants comprehensive examination.
